# A 21.4 pW Subthreshold Voltage Reference with 0.020 %/V Line Sensitivity Using DIBL Compensation

**DOI:** 10.3390/s23041862

**Published:** 2023-02-07

**Authors:** Louis Colbach, Taekwang Jang, Youngwoo Ji

**Affiliations:** 1 Department of Information Technology and Electrical Engineering, ETH Zurich, 8092 Zurich, Switzerland; 2 Department of Electronic Engineering, Hanbat National University, Daejeon 34158, Republic of Korea

**Keywords:** ultra-low-power, subthreshold voltage reference, line sensitivity, DIBL effect, temperature coefficient

## Abstract

This paper presents an ultra-low-power voltage reference designed in 180 nm CMOS technology. To achieve near-zero line sensitivity, a two-transistor (2-T) voltage reference is biased with a current source to cancel the drain-induced barrier-lowering (DIBL) effect of the 2-T core, thus improving the line sensitivity. This compensation circuit achieves a Monte-Carlo-simulated line sensitivity of 0.035 %/V in a supply range of 0.6 to 1.8 V, while generating a reference voltage of 307.8 mV, with 21.4 pW power consumption. The simulated power supply rejection ratio (PSRR) is −54 dB at 100 Hz. It also achieves a temperature coefficient of 24.8 ppm/°C in a temperature range of −20 to 80 °C, with a projected area of 0.003 mm^2^.

## 1. Introduction

With the growing demand for energy-efficient and portable devices, such as IoT sensors or biomedical applications, power and area constraints on integrated circuits (ICs) are becoming increasingly more significant due to the limited energy source and the system volume.

A voltage reference is a key building block for analog and mixed-signal ICs. It generates a well-defined and stable voltage, irrespective of variations in the supply voltage and temperature. A bandgap reference (BGR) is commonly adopted for such applications, thanks to its superior temperature coefficient (TC) and line sensitivity (LS), which indicate how the reference voltage varies depending on the temperature and supply voltage, respectively. Such BGRs are implemented with a proportional-to-absolute-temperature (PTAT) voltage that cancels the TC of a complementary-to-absolute-temperature (CTAT) voltage of a BJT [1,2,3]. For ultra-low-power miniaturized systems, however, the area, power consumption and supply requirements make it difficult to employ a BGR as a voltage reference. A viable alternative featuring a low supply voltage, pW-level power consumption and small area is a subthreshold voltage reference [4,5,6,7,8,9,10,11,12,13,14]. The two-transistor (2-T) reference in [4] achieves a fine LS of 0.044 %/V and a low TC of 54~176 ppm/°C, while consuming 5.5 pW and occupying a 1425 µm^2^ area. However, further improvement in the LS is limited due to the drain-to-source voltage dependency of the pull-up transistor implemented with the native transistor due to the drain-induced barrier-lowering (DIBL) effect. To improve the LS, [5] applies a self-regulating circuit to the 2-T reference and achieves a better LS of 0.0154 %/V. A self-biased voltage reference based on a self-cascoded MOSFET (SCM) is proposed in [6], improving the LS and TC at the cost of higher power consumption. Another self-biased voltage reference presented in [7] aims to make the biasing current supply independent, offering further LS improvements. However, the self-regulating circuit consumes much more power than the 2-T core (e.g., 1 nW in [5]), causing a significant power penalty. Furthermore, the self-biasing circuits require additional start-up circuits due to multiple operating points, increasing the circuit complexity and area requirement.

This paper proposes an ultra-low-power CMOS voltage reference that consumes 21.4 pW, with an enhanced compensation to achieve a LS of 0.020 %/V without using complex and power-hungry start-up and self-biasing circuits.

The remainder of this paper is structured as follows. Section 2 introduces the DIBL effect of the transistors in the proposed design and its impact on the LS. Section 3 describes the design and implementation of the proposed voltage reference. Section 4 briefly summarizes the design methodology. Section 5 presents the simulation results, and Section 6 concludes the paper.

## 2. DIBL Effect

DIBL is one of the prominent non-idealities in short-channel MOSFET devices and refers to the dependency of a MOSFET’s drain current $I_{DS}$ on its drain-source voltage, $V_{DS}$. This effect can be modeled as a reduction of the transistor’s threshold voltage, $V_{th}$, as a function of $V_{DS}$ [15]:

(1) $$V_{th}=V_{th0}-\lambda_{D}V_{DS},$$

where $V_{th0}$ is the threshold voltage as $V_{DS}$ approaches zero, and $\lambda_{D}$ is the DIBL effect factor. As the channel length decreases, $\lambda_{D}$ typically increases.

The subthreshold current of a MOSFET is given by:

(2) $$I_{DS}=I_{0}\cdot S\cdot \exp\left(\frac{V_{GS}-V_{th}}{mV_{T}}\right)\left(1-\exp\left(-\frac{V_{DS}}{V_{T}}\right)\right),$$

where $I_{0}=\mu C_{ox}\left(m-1\right)V_{T}^{2}$ and S=W/L, and μ, $C_{ox}$, m and $V_{T}$ are the carrier mobility, oxide capacitance, subthreshold slope factor and thermal voltage, respectively. Typically, the last term of (2), $\exp\left(-V_{DS}/V_{T}\right)$, becomes negligible when $V_{DS}$ is sufficiently higher than $V_{T}$ (e.g., $V_{DS}>6V_{T}$).

Therefore, by combining (1) and (2) while eliminating the last term of (2), we can obtain:

(3) $$I_{DS}=I_{0}\cdot S\cdot \exp\left(\frac{V_{GS}-V_{th0}+\lambda_{D}V_{DS}}{mV_{T}}\right).$$

**Figure 1:**
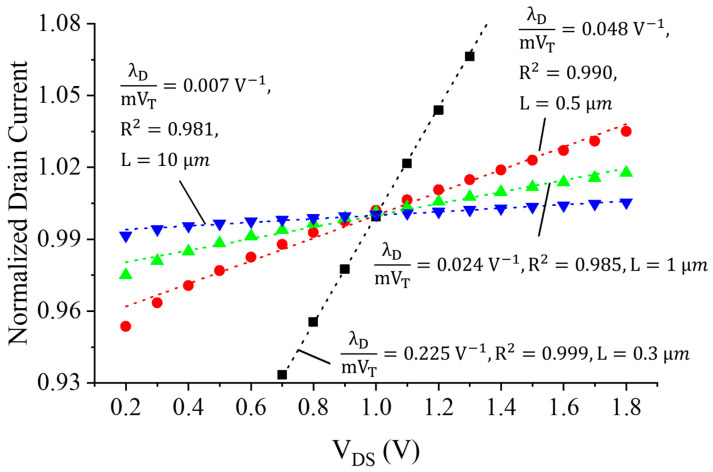
Simulated drain current of different-length PMOS transistors in 180 nm as a function of the drain-source voltage $V_{DS}$ with W/L=10 and $\left|V_{GS}\right|=100$ mV for all cases [7].

This clearly demonstrates the drain-source current’s dependence on $V_{DS}$ for a large $\lambda_{D}$, i.e., shorter channel lengths. Figure 1 shows the simulated drain current of a 3V PMOS device affected by the DIBL effect in a 180 nm CMOS technology. For $0.2\;V<V_{DS}<1.8\;V$, the drain current can be approximated as a linear function of $V_{DS},$ with a reasonable error less than 2 % (worst case), as indicated in [7]. This work models such a linear DIBL current using an effective aspect ratio $S_{eff}$, similarly to [7]:

(4) $$S_{eff}=S_{0}+\alpha \cdot V_{DS},$$

where $S_{0}$ is the W/L of the MOSFET, and α is the slope factor of the DIBL effect on the drain current, i.e., the slope of $I_{DS}$ as a function of $V_{DS}$.

## 3. Design and Analysis

### 3.1. Circuit Description

**Figure 2:**
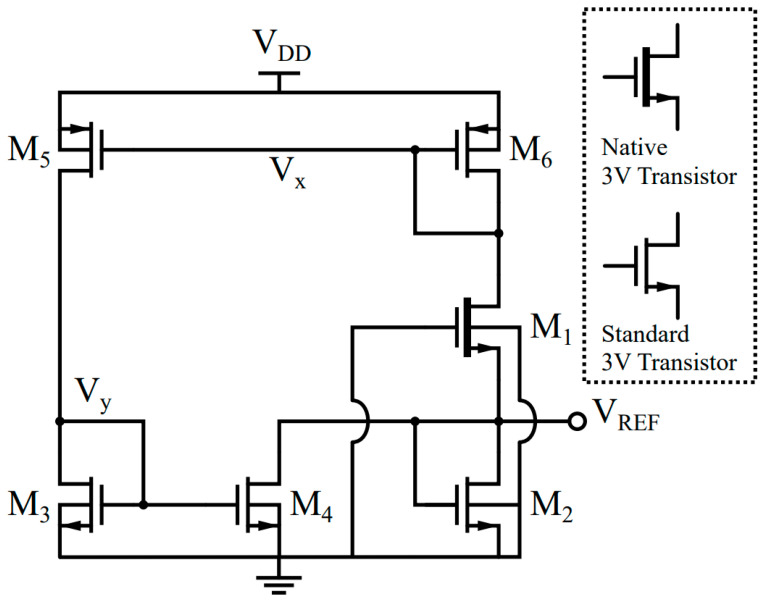
Schematic of the proposed voltage reference circuit.

A schematic of the proposed voltage reference is shown in Figure 2, and its sizing dimensions are listed in Table 1. It employs the 2-T voltage reference proposed in [4], formed by a native NMOS transistor (M1) stacked on top of a standard NMOS transistor (M2), generating a reference voltage $V_{REF},$ defined as a function of $V_{th2}-V_{th1}$. The native transistor has a low or near-zero $V_{th}$, which is much smaller than that of the standard transistor. To improve the supply independency, it performs compensation for the DIBL effect [7]. M5 and M6 generate currents dependent on the drain voltage due to the DIBL effect, which causes these currents to depend not only on the gate-source voltages $\left|V_{GS5,6}\right|$ of M5 and M6, but also on their drain-source voltages $\left|V_{DS5,6}\right|$. Due to the low resistance of the diode-configured transistor, M6, $\left|V_{DS6}\right|$ is almost constant along the supply change, whereas $\left|V_{DS5}\right|$ directly follows the supply change.

**Figure 3:**
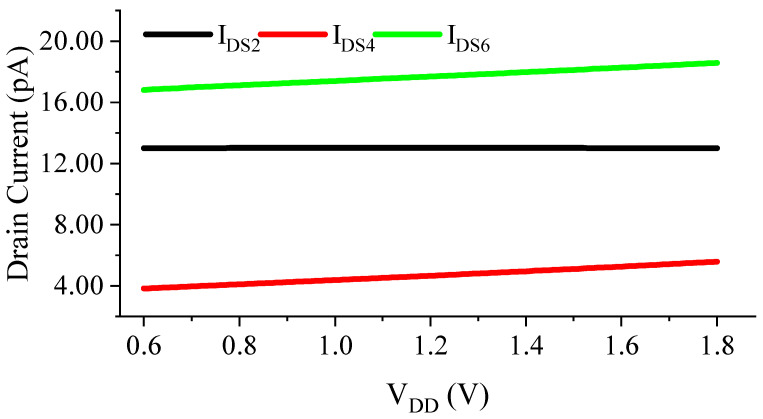
Simulated current biasing of M2.

Therefore, the current generated by M5 has a stronger supply dependency than the current generated by M6. The current mirror formed by M3 and M4 copies the current generated by M5 and subtracts it from the current generated by M1. Thus, the appropriate widths of M3 and M4 lead to a supply-independent current through M2 (Figure 3), resulting in a supply-independent reference voltage $V_{REF}$.

It is worth noting that the proposed voltage reference does not need additional branches to generate a biasing current. In addition, the proposed circuit only has one operating point due to the always-on leakage current of M1. Thus, it does not require an additional start-up circuit, either. The current through M1 and M6 is determined by the width and length of M1. Then, the current is mirrored to M5 and flows into M3. This current is again mirrored to M4, so the impedance of the diode-configured MOS transistors (M6 and M3) defines V_X_ and V_Y_. The current of M2 becomes $I_{DS,1}-I_{DS,4},$ and M2 converts the current to the reference voltage.

### 3.2. Minimum Supply Voltage

As mentioned above, (3) is only true for a large $V_{DS}$ (e.g., $6V_{T}$). Hence, the minimum supply voltage of the proposed circuit is decided by the output voltage, $V_{REF}$, plus two drain-source voltages, resulting in:

(5) $$V_{DD,min}\approx V_{REF}+6V_{T}+6V_{T}=V_{REF}+12V_{T}\approx 0.6\;V.$$

The low minimum-supply voltage is suitable for most low-power applications and contrasts with that of a BGR architecture, which is at least ~1.4 V ($V_{BGR}+\left|V_{DS}\right|$).

### 3.3. Temperature Coefficient

The proposed circuit operates robustly against temperature changes, as its 2-T structure, composed of M1 and M2, generates the output voltage as a function of the threshold voltage difference. Still, due to the additional current of the LS compensation circuit, the optimal transistor dimensions in the 2-T core for the lowest TC are different from the dimensions introduced in [4]. Assuming M5 and M6 are identical transistors, i.e., $W_{5}=W_{6}$ and $L_{5}=L_{6}$, we have $I_{0,5}=I_{0,6}$, $V_{th5}=V_{th6}$ and $m_{5}=m_{6}$, where $I_{0,k}$ represents $I_{0}$ of $M_{k}$. To account for the influence of the DIBL effect, $S_{5}$ and $S_{6}$ are modeled as effective aspect ratios, as described in (4), which entails $S_{eff,5}\neq S_{eff,6}$. Applying Kirchhoff’s current law at the output voltage, the current flowing into M2 can be written as:

(6) $$I_{DS,2}=I_{DS,1}-I_{DS,4}.$$

$I_{DS,1}$ and $I_{DS,2}$ can be re-written by using Equation (2), and $I_{DS,4}$ becomes $I_{DS,1}\frac{S_{5}}{S_{6}}\frac{S_{4}}{S_{3}}$ after passing through the two current mirrors. By arranging Equation (6), $V_{REF}$ can be obtained as:

(7) $$V_{REF}≅\frac{m_{1}V_{th2}-m_{2}V_{th1}}{m_{1}+m_{2}}+\frac{m_{1}m_{2}}{m_{1}+m_{2}}V_{T}\left(\ln\left(\frac{I_{0,1}S_{1}}{I_{0,2}S_{2}}\right)+\ln\left(1-\frac{S_{4}S_{5}}{S_{3}S_{6}}\right)\right).$$

Finally, assuming $L_{1}=L_{2}$ and setting the derivative over temperature equal to zero, the optimal transistor size ratio that cancels the temperature dependency of $V_{th}$ and $V_{T}=kT/q$ of the 2-T reference can be obtained as:

(8) $$\frac{W_{2}}{W_{1}}=\frac{I_{0,1}}{I_{0,2}}\left(1-\frac{S_{4}S_{5}}{S_{3}S_{6}}\right)exp\left(\frac{q}{k}\frac{m_{1}C_{Vth2}-m_{2}C_{Vth1}}{m_{1}m_{2}}\right),$$

where k is Boltzmann’s constant, and $C_{Vth1}$ and $C_{Vth2}$ are the first-order TCs of $V_{th1}$ and $V_{th2},$ respectively.

### 3.4. Line Sensitivity

The proposed circuit improves the LS by providing a constant biasing current through M2, which is independent of the supply voltage. By using (2), $V_{REF}$ is obtained as:

(9) $$V_{REF}=V_{th2}+m_{2}V_{T}\cdot \ln\left(\frac{I_{DS,2}}{I_{0,2}\cdot S_{2}}\right).$$

The currents $I_{5}$ and $I_{6}$ are subject to the DIBL effect, which can be found using (4). As shown in Figure 2, $\left|V_{GS5}\right|,|V_{GS6}|$ and $\left|V_{DS6}\right|$ are equal with each other. As in the derivation of (7), M5 and M6 are assumed to be identical transistors, leading to $W_{5}=W_{6}$, $L_{5}=L_{6}$, $I_{0,5}=I_{0,6}$, $S_{0,5}=S_{0,6}$, $V_{th0,5}=V_{th0,6}$ and $m_{5}=m_{6}$. Applying Kirchhoff’s current law at the output node leads to:

(10) $$I_{DS,2}=I_{DS,6}-\frac{S_{4}}{S_{3}}I_{DS,5}.$$

By substituting (4) and (9) into (10), $I_{DS,2}$ can be described as:

(11) $$I_{DS,2}=I_{0.6}exp\left(\frac{\left|V_{DS6}\right|-V_{th6}}{m_{6}V_{T}}\right)\left((S_{6}+\alpha_{6}\left|V_{DS6}\right|-\frac{S_{4}}{S_{3}}\left(S_{6}+\alpha_{5}\left|V_{DS5}\right|\right)\right).$$

Finally, (9) can be re-written as:

(12) $$\begin{array}{c} V_{REF}=V_{th2}+m_{2}V_{T}\ln\left(\frac{I_{0,6}S_{6}}{I_{0,2}S_{2}}\right)+\frac{m_{2}}{m_{6}}\left|V_{DS6}\right|-\frac{m_{2}}{m_{6}}V_{th6}+m_{2}V_{T}\ln\left(\left(1-\frac{S_{4}}{S_{3}}\right)+\alpha_{6}\left|V_{DS6}\right|-\frac{S_{4}}{S_{3}}\alpha_{5}\left|V_{DS5}\right|\right). \end{array}$$

**Figure 4:**
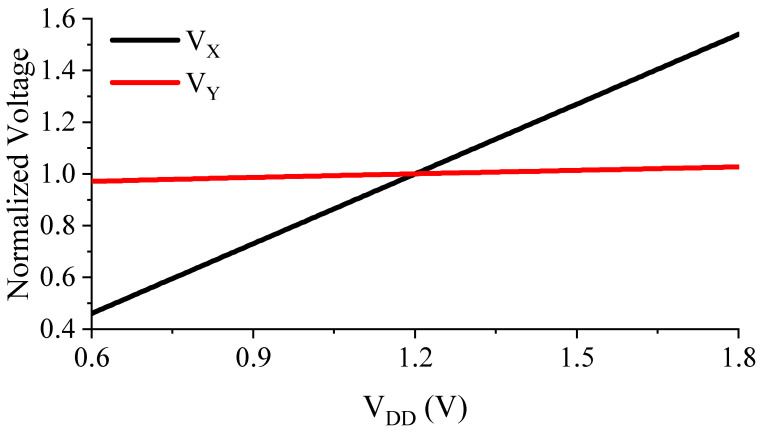
Linearity of the intermediate voltages $V_{X}$ and $V_{Y}$.

The intermediate node voltages, $V_{X}$ and $V_{Y}$ (Figure 2), show linear dependencies on the supply voltage, $V_{DD},$ in the given operating supply range of $0.6\;V<V_{DD}<1.8\;V$, as shown in the simulation results in Figure 4. This effect also influences $\left|V_{DS5}\right|$ and $\left|V_{DS6}\right|$, which can be modeled as follows:

$$V_{X}=V_{X0}+\gamma_{X}V_{DD}$$

(13) $$V_{Y}=V_{Y0}+\gamma_{Y}V_{DD}$$

where $V_{X0}$ and $V_{Y0}$ are the intermediate voltages $V_{X}$ and $V_{Y}$ at the minimum operating supply voltage $V_{DD,min}$, and $\gamma_{X}$ and $\gamma_{Y}$ are the slope factors of $V_{X}$ and $V_{Y}$, respectively. Combining (12) and (13) results in:

(14) $$\frac{\partial V_{REF}}{\partial V_{DD}}=\frac{m_{2}}{m_{6}}\left(1-\gamma_{X}\right)+\frac{m_{2}V_{T}\left(\alpha_{6}\left(1-\gamma_{X}\right)-\frac{S_{4}}{S_{3}}\alpha_{5}\left(1-\gamma_{Y}\right)\right)}{\left(1-\frac{S_{4}}{S_{3}}\right)+\alpha_{6}\left|V_{DS6}\right|-\frac{S_{4}}{S_{3}}\alpha_{5}\left|V_{DS5}\right|}$$

By making Equation $\left(14\right)$ equal to zero, the theoretical value for $S_{4}/S_{3}$ can be derived as:

(15) $$\frac{S_{4}}{S_{3}}=\frac{\left(1-\gamma_{X}\right)\left(1+\alpha_{6}\left|V_{DS6}\right|+m_{2}V_{T}\alpha_{6}\right)}{\left(1-\gamma_{Y}\right)m_{2}V_{T}\alpha_{5}+\left(1-\gamma_{X}\right)\left(1+\alpha_{5}\left|V_{DS5}\right|\right)}.$$

Equation $\left(15\right)$ can be simplified by using:

$$1-\gamma_{Y}=\frac{\partial \left|V_{DS5}\right|}{\partial V_{DD}}≅\frac{r_{o5}}{r_{o5}+\frac{1}{g_{m3}}}≅1\&1-\gamma_{X}=\frac{\partial \left|V_{DS6}\right|}{\partial V_{DD}}≅\frac{\frac{1}{g_{m6}}}{r_{o1}+\frac{1}{g_{m6}}}≅\frac{1}{g_{m6}r_{o1}},$$

and the simplified $S_{4}/S_{3}$ can be derived as:

(16) $$\frac{S_{4}}{S_{3}}≅\frac{1}{g_{m6}r_{o1}\lambda_{D5}+1},$$

where $g_{m}$ and $r_{o}$ represent the transconductance and output resistance, respectively.

**Figure 5:**
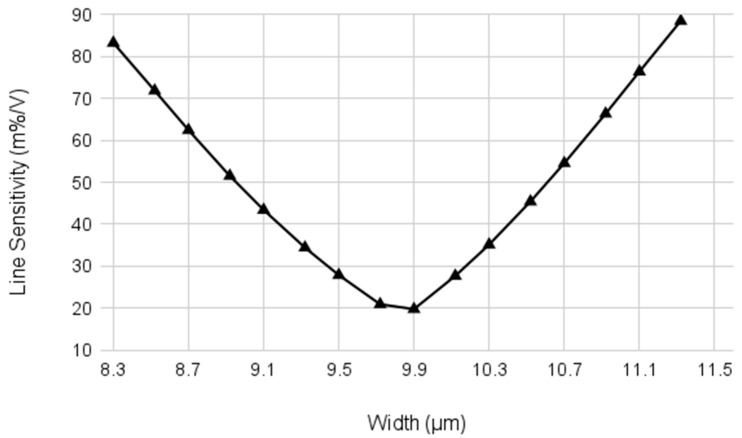
Line sensitivity depending on the width of M4.

From the initial design point in Equation $\left(16\right)$, the final optimum value can be found after several iterations. As in Equation (7), a change in the ratio $S_{4}/S_{3}$ leads to a small change in $V_{REF}$, which in turn affects $V_{X}$ and, thus, also $\left|V_{DS6}\right|$. Due to the DIBL effect of M6 and the fact that $\left|V_{GS5}|=|V_{DS6}\right|$, this then alters both biasing currents, requiring a re-adjustment of $S_{4}/S_{3}$. As shown in Figure 5, there is a single optimum point at which to achieve the minimum LS, which is only dependent on the DIBL coefficient of M5. After starting with the initial value of $S_{4}/S_{3}$, the next $S_{4}/S_{3}$ can be decided depending on the sign of $\partial V_{REF}/\partial V_{DD}$. If the sign is negative, it is over-compensated, and $S_{4}/S_{3}$ needs to be smaller, and vice versa.

### 3.5. Line Sensitivity

**Figure 6:**
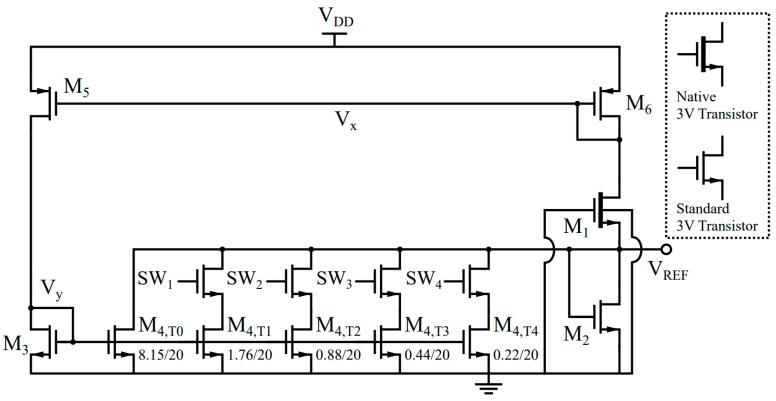
Schematic of the proposed circuit, including trimming for minimum line sensitivity.

**Figure 7:**
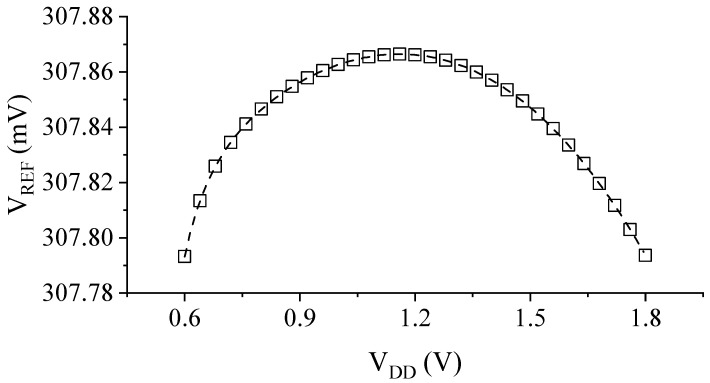
Simulated variation of $V_{REF}$ versus $V_{DD}$ in the suggested operating range of $0.6\;V<V_{DD}<1.8\;V$.

To deal with the impact of possible process and mismatch variations on the LS, the proposed design adopts a trimming block, as shown in Figure 6. The trimming circuit allows the digital adjustment of the bottom current mirror ratio $S_{4}/S_{3}$, which will adapt the amount of the compensation current. To cover the most relevant range of possible process variations with sufficient resolution, a 4-bit trimming code is employed to control switches SW1 to SW4, adjusting the mirroring ratio. The default trimming code is 1000, which results in the LS shown in Figure 7 at the nominal condition without any mismatch. After fabrication, the reference voltages at the minimum and maximum supply voltages can be obtained and used to determine the best trimming code by finding the code that minimizes the difference between the voltages. When the reference voltage increases as the supply voltage becomes larger, the trimming code also needs to be increased to make a larger compensating current, and vice versa.

## 4. Design Methodology

- *Determining the dimensions of M5 and M6*: The current mirroring ratio between M3 and M4 needs to be small ($S_{4}/S_{3}<1$) to reduce the DC bias current of M4, so that the output TC is rarely affected by I_DS,4_. At the same time, to make the supply-dependent current of M4 match with that of M1 while considering such a small mirroring ratio (Figure 3), the length of the PMOS current mirror (M5) needs to be short to create a relatively large supply-dependent current. The width of M6 is found to set $\left|V_{GS6}\right|=\left|V_{DS6}\right|\approx 0.2\;V$ and to achieve ${VDD}_{min}=0.6\;V$.
- *Determining the optimal dimensions of the 2-T reference*: The temperature sensitivity of the proposed circuit is mainly provided by the 2-T reference. M1 and M2 should, thus, be sized to minimize the TC. This step only considers M1, M2 and M6, i.e., M4 is disconnected from the output node. The length of M1 is chosen to be large enough, such that these transistors mitigate the DIBL effect and obtain a better power supply rejection ratio (PSRR). The width of M1 is set by considering the power budget. Finally, the dimension of M2 is found by using the optimum 2-T ratio found in (8) to minimize the TC of *V_REF_*.
- *Determining the dimensions of M3 and M4*: The channel lengths of M3 and M4 should again be chosen to be large enough for better matching. The proper ratio between M3 and M4 determines the LS of the circuit to cancel the DIBL between M1 and M5, as shown in Figure 3.
- *Re-optimizing M2 and M4*: The additional current of M4 alters the optimum ratio between M1 and M2 found in (8). It is, thus, suggested to slightly re-adjust the size of M2, which in turn might require another change in M4 to also re-optimize for the lowest LS. This optimization loop can be continued until both the TC and LS settle on satisfactory values.

## 5. Simulation Results

In this section, we present the SPICE simulation results of the proposed voltage reference scheme shown in Figure 2 and Table 1. To compare the results, the 2-T voltage reference in [4] is also simulated under the same conditions. The dimensions for the transistors in the 2-T core are determined using the values in [4], and the width of the bottom transistor is slightly changed to achieve the minimum TC in the given simulation environment.

**Figure 8:**
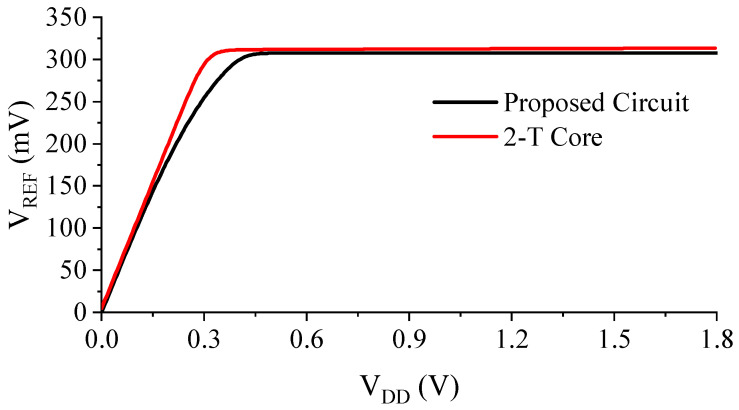
Simulated output voltage of the proposed voltage reference in comparison with the conventional 2-T reference.

Figure 8 shows the generated reference voltage $V_{REF}$ as a function of the supply voltage $V_{DD}$ in the range of 0–1.8 V. As shown in Figure 7, the proposed design generates a constant $V_{REF}$ of ~307.8 mV with a LS of only 0.020 %/V, which is evaluated using the following equation:

(17) $$LS=\frac{\Delta V_{REF}}{\Delta V_{DD}\cdot V_{REF,AVG}}\cdot 100\%$$

**Figure 9:**
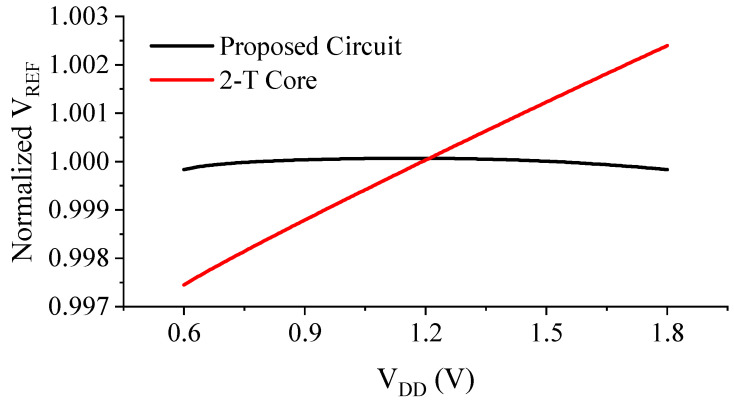
Normalized and magnified output voltages of the proposed design and the conventional 2-T reference.

Figure 8 and Figure 9 show the output voltage of the proposed design in comparison with the conventional 2-T reference. Although *V_DD,min_* is about 100 mV higher due to the additional M6 connected to the drain of M1, the LS is improved by 18-fold, thanks to the proposed compensation scheme (Figure 9).

**Figure 10:**
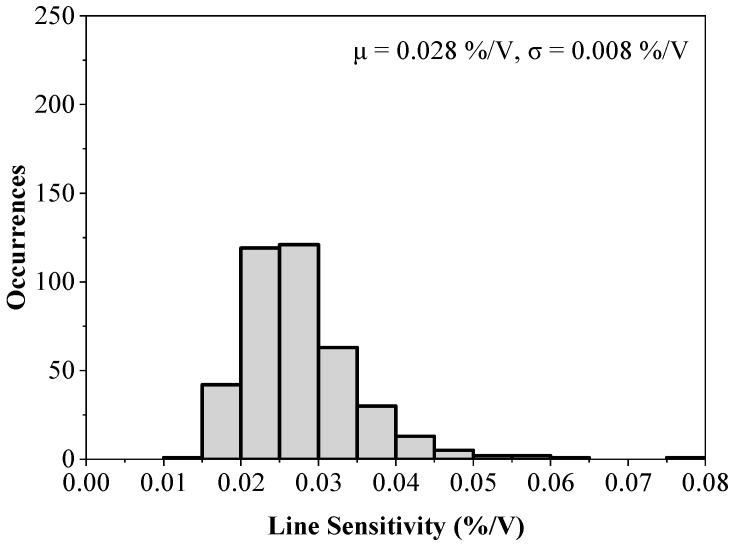
Monte Carlo simulation results for the line sensitivity of the circuit presented in Figure 2 (400 runs).

**Figure 11:**
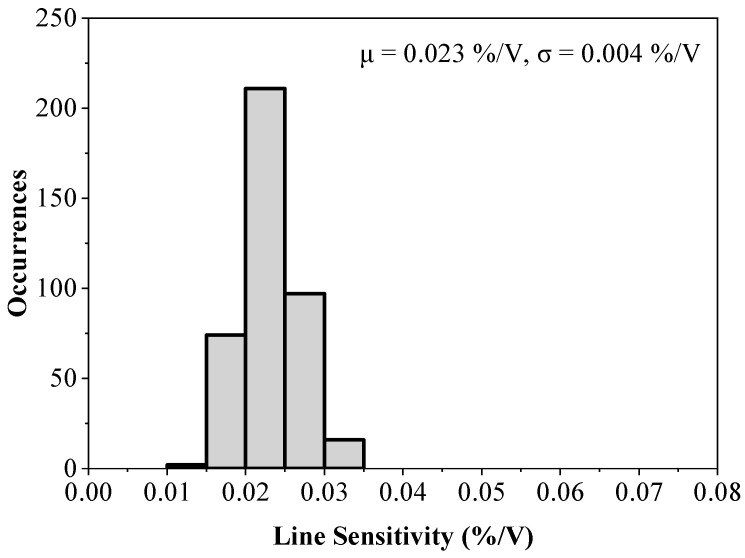
Monte Carlo simulation results for the line sensitivity of the circuit presented in Figure 6 using the optimal trimming code for each run (400 runs).

Figure 10 shows the LS of the untrimmed circuit from a 400-point Monte Carlo simulation to verify the performance of the proposed design under the device mismatch. The worst case LS is 0.08 %/V, which is about a 4x increase compared to the case without any variations. Re-running the same Monte Carlo simulation after the 4-bit trimming leads to the results shown in Figure 11. It can be seen that less than 0.035 %/V LS is achieved, validating the superior performance of the proposed voltage reference.

**Figure 12:**
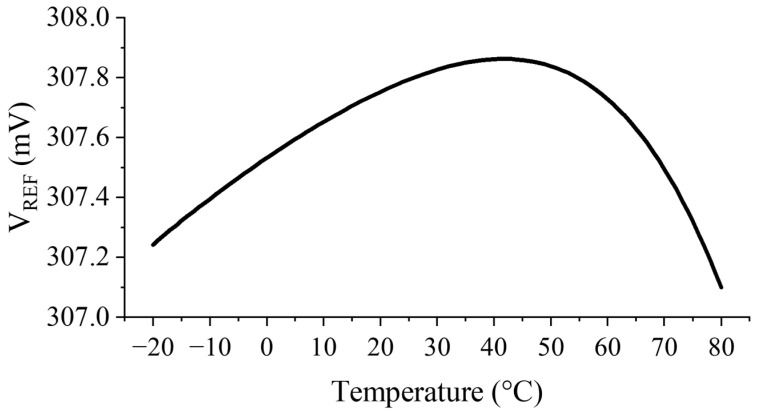
Simulated temperature variation of $V_{REF}$.

The simulated temperature sensitivity between −20 and 80 °C of the proposed circuit is shown in Figure 12. The TC in ppm/°C is given by the following expression:

(18) $$TC=\frac{\Delta V_{REF}}{\Delta T\cdot V_{REF}\left(27{}^{\circ}C\right)}\cdot 10^{6}$$

In the suggested operating temperature range, a simulated TC of 24.8 ppm/°C is obtained at a supply voltage of 0.6 V.

**Figure 13:**
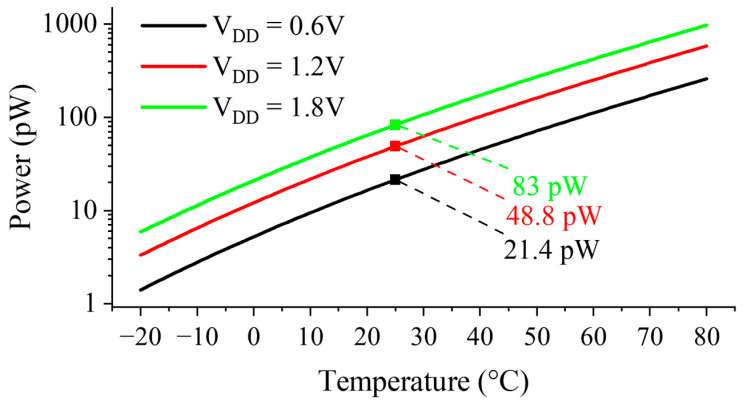
Simulated power consumption.

Figure 13 shows the simulated power consumption of the proposed design. At room temperature, the proposed voltage reference requires 21.4 pW at a supply of 0.6 V and 83 pW at the maximum operating supply of 1.8 V. The highest simulated power consumption occurs at $V_{DD}=1.8\;V$ and T = 80 °C, reaching 974 pW.

**Figure 14:**
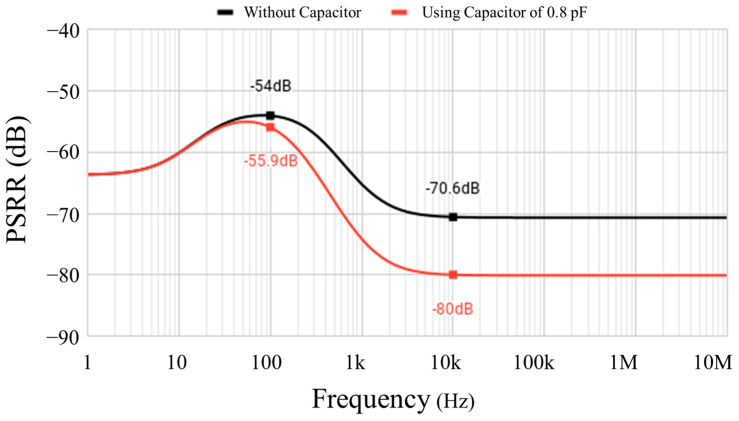
Simulated PSRR at 25 °C and $V_{DD}=0.6\;V$.

Figure 14 presents the PSRR of the proposed design, with and without a capacitor. The capacitor can be located above the active area, so that it does not occupy an additional space. At a frequency of 100 Hz, a PSRR of −54 dB can be obtained. At frequencies above 8 kHz, the PSRR plateaus at around −70.6 dB. With an additional capacitor of 0.8 pF, a PSRR as low as −80 dB can be attained above 10 kHz.

Table 2 summarizes the performance of the proposed voltage reference and provides a comparison to other references published in recent years, notably the 2-T reference [4] that is employed as the base reference. By performing a simple 4-bit trimming using the results of the 400-point Monte Carlo simulation, the proposed design achieves a LS of 0.035 %/V, the lowest among the sub-nW voltage references. In addition, compared to another design adopting DIBL compensation [7], the proposed design occupies 10-fold less area.

## 6. Conclusions

This paper presented an ultra-low-power CMOS voltage reference with an LS improvement technique using DIBL-effect cancellation without additional self-bias feedback loops or start-up circuits. The simulation results in 180 nm CMOS show that the circuit generates a reference voltage of 307.8 mV, while consuming only 21.4 pW of power at nominal conditions. According to 400-point Monte Carlo simulations, the worst case LS of 0.035 %/V is achieved across numerous process and mismatch variations after a 4-bit trimming circuit. The simulated PSRR is -54 dB at the worst condition of 100 Hz and at a minimum operating supply of 0.6 V. The proposed design is well-suited for energy-constrained systems, such as battery-operated IoT devices, thanks to its ultra-low power and superior accuracy characteristics.

## Figures and Tables

**Table 1:** MOSFET dimensions of the proposed circuit.

Transistor	Width (µm)	Length (µm)	Current (pA)
M1 (Native)	3.2	20	17
M2	10.2	20	13
M3	50	20	19
M4	9.91	20	4
M5	100	0.3	19
M6	100	0.3	17

**Table 2:** Performance summary and comparison to other works.

	This Work *	[4]	[5]	[6]	[7]	[8]	[9]	[10]	[11]	[12]	[13]	[14]
Technology (nm)	180	180	180	180	180	180	130	180	40	180	180	65
*V_DD_* (V)	0.6~1.8	0.5~3.6	0.4~1.8	0.45~3.3	0.34~1.8	1.4~3.6	0.3~1.2	1.2~2.2	1.2~1.8	0.9~1.8	0.25~1.8	0.4~1.2
*V_REF_* (V)	0.3078	0.3284	0.151	0.2566	0.1479	1.25	0.026	0.9862	0.8	0.261	0.094	0.3428
Temp. Range (°C)	−20~80	−20~80	−40~125	0~120	0~100	0~100	−25~125	−40~85	−40~90	−40~130	0~120	−40~60
TC (ppm/°C)	24.8	115.3	89.83	72.4	14.8	31	208	86	3	62	265	252.2
LS (%/V)	0.020	0.044	0.163	0.15	0.019	0.31	0.188	0.38	0.028	0.013	0.16	0.47
Worst LS (%/V)	0.035	N/A	N/A	N/A	0.039 *	N/A	N/A	N/A	N/A	N/A	0.31	N/A
# of Samples	400	14	16	5	39 400 *	60	5	60	200	15	30	38
PSRR @ 100 Hz (dB)	−54	−49	−55	−43.9	−63	−41	−67.3 *	−42	−71.7	−73.5	−70	N/A
Power @ 25 °C (pW)	21.4	5.5	1000	147	48	33.6	40	114	9600000	1800	5.4	0.42 @ 20 °C
Area (mm^2^)	0.003	0.001425	0.005	0.002	0.0332	0.0025	0.0006	0.00488	N/A	0.0059	0.0022	0.00010

* Simulation Result.

## Data Availability

Not applicable.
